# Prognostic Role of Beta‐2 Microglobulin in Diffuse Large B‐Cell Lymphoma: Systematic Review and Meta‐Analysis of Observational Studies

**DOI:** 10.1002/cnr2.70416

**Published:** 2025-11-29

**Authors:** Hamideh Feiz Disfani, Mahsan Ramezani, Milad Moradi, Mostafa Kamandi, Molood Foogerdi, Vajiheh Sadat Saghi, Rasoul Shavaleh, Kazem Rahmani

**Affiliations:** ^1^ Department of Emergency Medicine School of Medicine, Mashhad University of Medical Sciences Mashhad Iran; ^2^ Department of Emergency Medicine School of Medicine, Neyshabur University of Medical Sciences Neyshabur Iran; ^3^ Nano Drug Delivery Research Center, Health Technology Institute, Kermanshah University of Medical Sciences Kermanshah Iran; ^4^ Department of Medical Physics School of Medicine, Iran University of Medical Sciences Tehran Iran; ^5^ Hematologist‐Oncologist, Department of Internal Medicine Faculty of Medicine, Mashhad University of Medical Sciences Mashhad Iran; ^6^ Department of Emergency Medicine Faculty of Medicine, Birgand University of Medical Sciences Birjand Iran; ^7^ Torbat‐e Jam University of Medical Sciences Torbat‐e Jam Iran; ^8^ Department of Epidemiology School of Public Health, Iran University of Medical Sciences Tehran Iran; ^9^ Non‐Communicable Disease Center, Torbat‐e Jam University of Medical Sciences Torbat‐e Jam Iran

**Keywords:** beta‐2 microglobulin, DLBCL, non‐Hodgkin's lymphoma, prognosis

## Abstract

**Background:**

Evidence indicates that beta‐2 microglobulin (β2M) plays a prognostic role in patients with diffuse large B‐cell lymphoma (DLBCL).

**Aim:**

However, due to controversies among previous studies, this meta‐analysis was conducted to evaluate the prognostic role of β2M in DLBCL patients.

**Methods and Results:**

Relevant studies were identified through a systematic search using specific keywords in PubMed/Medline, Scopus, and Web of Science databases. Studies that provided complete information on the hazard ratio with a 95% confidence interval for β2M with respect to overall survival (OS) and progression‐free survival (PFS) in multivariate models were included in the meta‐analysis. A total of 30 studies, encompassing 25 128 patients, were included in this meta‐analysis. The pooled analysis demonstrated a significant association between elevated β2M levels and poor OS in DLBCL patients (HR _Pooled_: 1.65, 95% CI: 1.45–1.88, *p*‐value < 0.01). Similarly, increased β2M levels were significantly associated with lower PFS in DLBCL patients (HR _Pooled_: 1.54, 95% CI: 1.39–1.70, *p*‐value < 0.01).

**Conclusion:**

The available evidence suggests that β2M serves as an independent prognostic marker with significant effects on OS and PFS in DLBCL patients. Elevated serum β2M levels are associated with poor prognosis and worse OS and PFS outcomes in DLBCL patients.

## 1 Introduction

Diffuse large B‐cell lymphoma (DLBCL) is one of the most common pathological forms of lymphoma, characterized by high heterogeneity and poor prognosis. It accounts for approximately 40% of all non‐Hodgkin's lymphomas (NHLs) [1, 2, 3]. Unlike other solid malignancies, the survival rate of DLBCL patients varies significantly due to its diverse clinical course and outcomes. The 10‐year survival rate for DLBCL is around 40%, with over 50% of patients responding to treatment regimens such as R‐CHOP (rituximab, cyclophosphamide, doxorubicin, vincristine, and prednisolone) and R‐CHOP‐like therapies [4, 5, 6, 7]. However, the relapse risk remains approximately 40%, and patients with advanced DLBCL generally experience poor prognosis and low survival rates [8].

The International Prognostic Index (IPI) and age‐adjusted International Prognostic Index (aaIPI) is a widely used clinical tool for risk stratification in DLBCL patients, assisting in evaluating treatment responses and clinical outcomes [9, 10]. The aaIPI is a streamlined clinical tool specifically developed to predict prognosis in patients with DLBCL, particularly in those under 60 years of age [11]. Although aaIPI offers simplicity and ease of use, the original IPI encompasses a wider array of prognostic variables, resulting in superior stratification accuracy and predictive performance in DLBCL patients [12].

Despite its widespread application, IPI does not account for certain intrinsic biological factors affecting risk classification. To improve clinical prognostic accuracy, risk classification models such as revised IPI (R‐IPI), biological IPI (B‐IPI), and National Comprehensive Cancer Network IPI (NCCN‐IPI) have been introduced [13, 14, 15]. However, despite these advancements, studies indicate that current prognostic indices still lack sufficient accuracy for clinical application in identifying high‐risk cases [16]. In recent years, various biological, molecular, and genetic markers with prognostic value have been identified in DLBCL patients. However, the clinical application of certain prognostic markers, such as gene signatures, is limited due to their complexity and high cost, making simpler and more affordable markers more desirable [17, 18]. Emerging evidence suggests that tumor‐related and immune‐related biomarkers provide valuable prognostic information [19]. Beta‐2 microglobulin (β2M) has recently been recognized as a hematologic marker in DLBCL, derived directly from a complete blood count (CBC). Studies suggest that β2M may have a prognostic role in DLBCL patients [20, 21, 22]. β2M is a non‐glycosylated protein produced by all nucleated cells except trophoblastic erythrocytes and is a key component of the light chain of the human leukocyte antigen (HLA) Class I complex. It is primarily metabolized and excreted by the kidneys [21, 22, 23] and is found on the membrane of nearly all nucleated cells, particularly white blood cells, which serve as its main source [24, 25].

Although several individual studies have evaluated the prognostic value of β2‐microglobulin (β2M) in DLBCL, their findings remain inconsistent and inconclusive. Some have suggested a strong prognostic role for β2M in predicting overall survival (OS) and progression‐free survival (PFS) [19, 21, 22, 26, 27], while others found no significant association [28, 29, 30]. To date, no comprehensive and up‐to‐date meta‐analysis has systematically synthesized these findings to clarify the prognostic significance of β2M in DLBCL patients. This study distinguishes itself by conducting a rigorous and updated meta‐analysis incorporating recent high‐quality studies, aiming to resolve these discrepancies. Furthermore, this analysis explores potential subgroup effects and sources of heterogeneity, providing new insights into the clinical utility of β2M as a cost‐effective and widely accessible prognostic biomarker. The findings of this research may contribute to refining risk stratification strategies and guiding more personalized treatment decisions in patients with DLBCL.

## 2 Materials and Methods

This systematic review and meta‐analysis was conducted based on the Epidemiological Checklist for Observational Studies in Meta‐Analysis [31] and the PRISMA checklist [32]. The systematic review protocol was registered in the international PROSPERO database (Registration Code: CRD42023423027), aiming to assess the prognostic role of β2M in patients with DLBCL.

### 2.1 Search Strategy

A literature search was conducted to identify published studies up to February 1, 2025, using study keywords based on Medical Subject Headings (MeSH) in the PubMed/Medline, Web of Science Core Collection, and Scopus databases. The search terms used in this meta‐analysis included:

(Beta 2‐Microglobulin) OR (β 2‐Microglobulin) OR (β2M) OR (Beta 2M) AND (DLBCL) OR (Diffuse large B‐cell lymphoma) OR (Diffuse large B cell) OR (B cell lymphoma) OR (lymphoma) OR (leukemia) (Supporting Information S1).

The search was independently conducted by two researchers. The titles and abstracts of the retrieved articles were screened, and irrelevant studies were excluded. The full texts of the remaining articles were then reviewed based on the inclusion and exclusion criteria. Additionally, reference lists of the selected articles were manually searched to identify further relevant studies.

### 2.2 Eligibility Criteria

All observational studies (prospective and retrospective cohort and case–control studies) evaluating the association between β2M and survival/prognosis in DLBCL patients were included in the analysis. There were no time restrictions, and only studies published in English were considered.

### 2.3 Inclusion Criteria

- Original studies published in English.
- Patients diagnosed with DLBCL, confirmed based on pathological criteria.
- Studies evaluating β2M levels.
- Studies reporting HR for OS and PFS obtained from multivariate Cox regression analysis.

### 2.4 Exclusion Criteria

- Animal studies.
- Studies published in languages other than English and duplicate studies.
- Case reports, case series, letters, or correspondence articles.
- Studies on patients with HIV/AIDS, immunosuppressed individuals, or those with autoimmune diseases.
- Studies lacking adjustment for confounding factors in OS and PFS analysis or those that did not provide sufficient data.

### 2.5 Data Extraction and Quality Assessment

**FIGURE 1:**
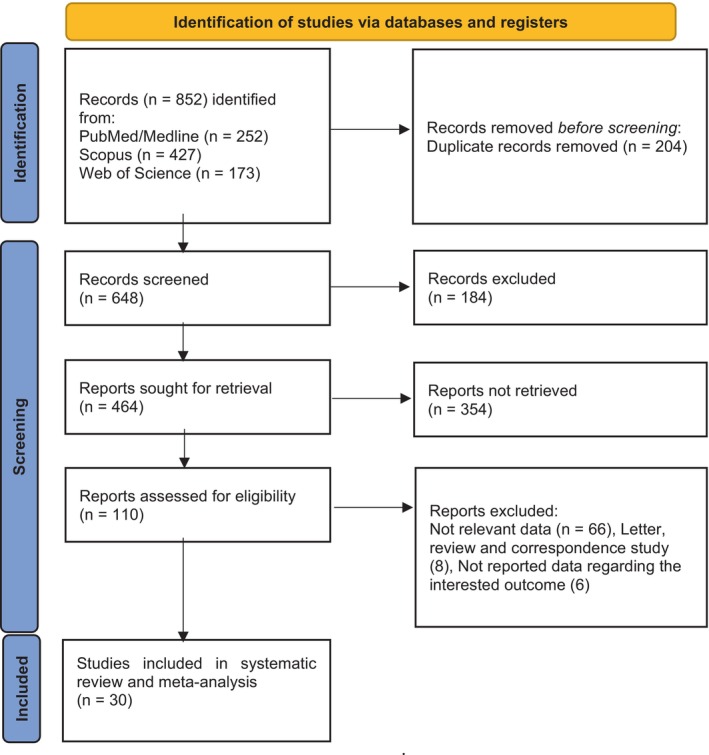
PRISMA flow diagram of the studies included in this meta‐analysis.

Two independent researchers (K.R. and R.S.H.) selected relevant articles by screening their titles and abstracts. Studies that did not meet the inclusion criteria were excluded (Figure 1). Any discrepancies were discussed until consensus was reached. The extracted data included author name, year of publication, study location, study type, median age and follow‐up duration, gender ratio (M/F), survival outcomes analyzed (OS and PFS), sample size, treatment type, and adjusted hazard ratios (HR) with 95% confidence intervals. Information on β2M measurement methods and cut‐off values was also recorded. PFS was defined as the time from treatment initiation until disease progression, relapse, or death from any cause. OS was defined as the time from the start of medical treatment until death or the end of follow‐up for any reason.

To evaluate study quality, two researchers (H.F.D. and M.R.) independently applied the Newcastle‐Ottawa Scale (NOS) [33]. Any disagreements were reviewed and discussed. Studies with NOS ≥ 7 were considered high quality.

### 2.6 Statistical Analysis

Statistical analysis was conducted using Stata 17 (Corp, College Station, TX, USA). Heterogeneity between studies was assessed using *I* ^2^, Chi^2^, and Tau^2^ statistics. An *I* ^2^ > 50% and *p* < 0.1 indicated significant heterogeneity, prompting the use of a Der‐Simonian and Laird (D + L) random‐effects model to calculate pooled HR with 95% CI. If studies were homogeneous, a fixed‐effects model using the inverse variance method was applied for weighting and pooling HR with 95% CI. Sensitivity analysis was performed using a leave‐one‐out approach to assess the stability of the results. Subgroup analysis was conducted to identify potential sources of heterogeneity. Publication bias was visually inspected using an enhanced contour funnel plot, while statistical tests, including Egger's test and Begg's test, were used for further assessment. A significance level of *p*‐value < 0.05 was considered statistically significant.

## 3 Results

In total, 30 studies were included in this meta‐analysis based on keyword searches [16, 19, 20, 21, 22, 26, 27, 28, 29, 30, 34, 35, 36, 37, 38, 39, 40, 41, 42, 43, 44, 45, 46, 47, 48, 49, 50, 51, 52, 53] (Figure 1). Among the included 27 studies with a combined sample size of 17 407 patients examined OS [19, 20, 21, 22, 26, 27, 28, 29, 30, 34, 35, 36, 37, 38, 39, 40, 41, 42, 43, 46, 47, 48, 49, 50, 51, 52, 53], and 13 studies with a combined sample size of 7721 patients analyzed PFS [16, 19, 21, 28, 29, 35, 41, 44, 45, 47, 48, 51, 53].

Regarding study design, 28 studies were retrospective [16, 19, 20, 21, 22, 26, 27, 28, 30, 34, 35, 36, 37, 38, 39, 40, 41, 43, 44, 45, 46, 47, 48, 49, 50, 51, 52, 53] and 2 studies were prospective [29, 42]. In terms of geographical distribution, 11 studies (36.67%) were conducted in China, 7 studies (23.34%) were conducted in South Korea, 4 studies (13.33%) were conducted in Japan, 3 studies (10%) were conducted in Spain, and 5 studies (16.66%) were conducted in other countries such as the USA, Iraq, Denmark, Austria, and Latin America. The studies were conducted between 2013 and 2024 (Table 1).

**TABLE 1:** Summary of the studies included in meta‐analysis.

Author	Year	Country	Description	Type of study	Median/mean age	Gender ratio (M/F)	Follow up time (month)	Survival analysis	Sample size	Treatment	HR (95% CI)
De Zhou [52]	2013	China	β2M > UNL (2200 μg/L)	Retrospective	54	1.29	19	OS	227	CHOP and R‐CHOP	0.978 (0.509–1.88)
Kazuho Miyashita [45]	2014	Japan	β2M > UNL (1.75 μg/mL) (1.4‐times upper normal limit)	Retrospective	63	1.31	—	PFS	319	R‐CHOP	2.11 (1.04–4.31)
Seyoung Seo [48]	2014	South Korea	β2M > UNL (2.5 mg/L)	Retrospective	—	—	—	PFS, OS	834	R‐CHOP	PFS: 1.9 (1.3–2.6) OS: 2.1 (1.4–3)
Thomas Melchardt [43]	2014	Austria	β2M > UNL (3 mg/L)	Retrospective	65.3	1.11	51	OS	499	R‐CHOP and like R‐CHOP regime	2.161 (1.168–3.998)
Kazuho Miyashitaa [44]	2015	Japan	β2M > UNL (1.75 μg/mL)	Retrospective	63	1.31	50.1	PFS	319	R‐CHOP	2.09 (1.02–4.28)
Yiming Chen [21]	2016	USA	β2M > 1 UNL	Retrospective	58	1.06	60.4	PFS, OS	817	R‐CHOP, R‐hyperCVAD/R‐MA, REPOCH	PFS: 1.69 (1.08–2.64) OS: 3.38 (1.75–6.55)
Seyoung Seo [47]	2016	South Korea	β2M > UNL (2.5 mg/L)	Retrospective	58	1.32	47.6	PFS, OS	833	R‐CHOP	PFS: 1.7 (1.29–2.24) OS: 2 (1.47–2.75)
Tzu‐Hua Chen‐Liang [36]	2017	Spain	β2M > UNL (2.5 mg/L)	Retrospective	61	0.99	25	OS	203	R‐CHOP	2.2 (1–4.5)
Junshik Hong [38]	2017	South Korea	β2M > 1 UNL	Prospective	60	1.32	55	OS	439	R‐CHOP	1.55 (1.02–2.35)
Yusuke Kanemasa [22]	2017	Japan	β2M > UNL (3.2 mg/L)	Retrospective	69	1.21	37	OS	274	R‐CHOP	2.98 (1.64–5.45)
Jihoon Kang [42]	2017	South Korea	β2M > UNL (2.5 mg/L)	Prospective	57	1.23	43.3	OS	621	R‐CHOP	1.39 (0.951–2.04)
Carlos Montalban [20]	2017	Spain	β2M categorized into normal and high (> 1)	Retrospective	60	1.05	59	OS	1085	R‐CHOP	1.44 (1.11–1.86)
Hyeun‐Su Im [39]	2018	South Korea	β2M > UNL (2.4 μg/mL)	Retrospective	52	1.49	14.1	OS	117	Salvage chemotherapy and autologous hematopoietic stem cell transplantation (ASCT)	1.9 (1.1–3.2)
Norihito Inoue [40]	2018	Japan	β2M > UNL (2.23 mg/L)	Retrospective	65		58.5	OS	647	R‐CHOP	1.86 (1.18–2.93)
Wenqin Yue [50]	2018	China	β2M > UNL (2.8 mg/L)	Retrospective	58	1.25	34	OS	335	Pretreatment	1.598 (1.069–2.387)
Pan Zhao [51]	2018	China	β2M > UNL (2.65 mg/L)	Retrospective	58	1.51	47	PFS, OS	309	R‐CHOP	PFS: 1.327 (0.917–1.92) OS: 1.499 (1.012–2.219)
Ying Han [37]	2019	China	β2M categorized into normal and elevated	Retrospective	53	1.23	57	OS	748	R‐CHOP and like R‐CHOP regime	1.75 (1.28–2.38)
Luis Villela M [49]	2019	Latin America	β2M > UNL (4 μg/dL)	Retrospective		1.08	32	OS	525	R‐CHOP	1.4 (1.02–1.94)
Leyre Bento [16]	2020	Spain	β2M categorized into normal and high according to the initial cut off value	Retrospective	64	1.02	55	PFS	992	R‐CHOP	1.44 (1.1–1.9)
Da Jung Kim [29]	2020	South Korea	β2M > UNL (3.5 mg/L)	Retrospective	63	0.97		PFS, OS	312	R‐CHOP	PFS: 1.29 (0.77–2.18) OS: 1.28 (0.73–2.25)
Jin Roh [46]	2020	South Korea	The prognostic value of serum β2M in DLBCL as a continuous variable was confirmed without determining a specific cut‐off point.	Retrospective	59	1.35	59	OS	204	R‐CHOP	1.2 (1.1–1.31)
Jun Cai [34]	2021	China	β2M > UNL (3.2 mg/L)	Retrospective	—	1.3	81.3	OS	1406	R‐CHOP	1.03 (0.817–1.298)
Kawa Muhamedamin Hasan [28]	2021	Iraq	β2M > UNL (3 mg/L)	Retrospective	51	2.09	35	PFS, OS	136	R‐CHOP	PFS: 1.632 (0.748–3.56) OS: 0.976 (0.486–1.959)
Haizhu Chen [19]	2022	China	β2M categorized into normal and elevated	Retrospective	53	1.24	85.2	PFS, OS	701	R‐CHOP and like R‐CHOP regime	PFS: 1.543 (1.181–2.016) OS: 1.41 (1.04–1.913)
Weiling Zhou [53]	2022	China	β2M > UNL (2.8 μg/mL)	Retrospective	60.71	1.01	35.8	PFS	1767	CHOP and R‐CHOP	PFS: 1.36 (1.12–1.66) OS: 1.39 (1.14–1.7)
Fenglian Jing [41]	2023	China	The prognostic value of serum β2M in DLBCL as a continuous variable was confirmed without determining a specific cut‐off point.	Retrospective	54.8	1.26	—	PFS, OS	201	R‐CHOP and R‐CHOP like	PFS: 1.904 (1.06–3.421) OS: 1.933 (0.927‐ 4.03)
Yang Chen [35]	2023	China	β2M categorized into the normal and elevated	Retrospective	60	1.21	35	PFS, OS	181	R‐CHOP	PFS: 1.801 (0.837–3.875) OS: 2.963 (1.266–6.937)
Jelena Jelicic [27]	2024	Denmark	β2M > 1 UNL	Retrospective	68	1.29	59.5	OS	3232	CHOP and R‐CHOP	2.137 (1.875–2.093)
Mengdi Wan [26]	2024	China	β2M > UNL (3.5 mg/L)	Retrospective	—	1.09	—	OS	402	CHOP, R‐CHOP and R‐DA‐EPOCH	3.301 (1.987–5.482)
Zanzan Wang [30]	2024	China	β2M > UNL (3.05 mg/L)	Retrospective	62	1.02	44	OS	352	R‐CHOP	1.267 (0.62–2.58)

### 3.1 Relationship Between OS and β2M

**FIGURE 2:**
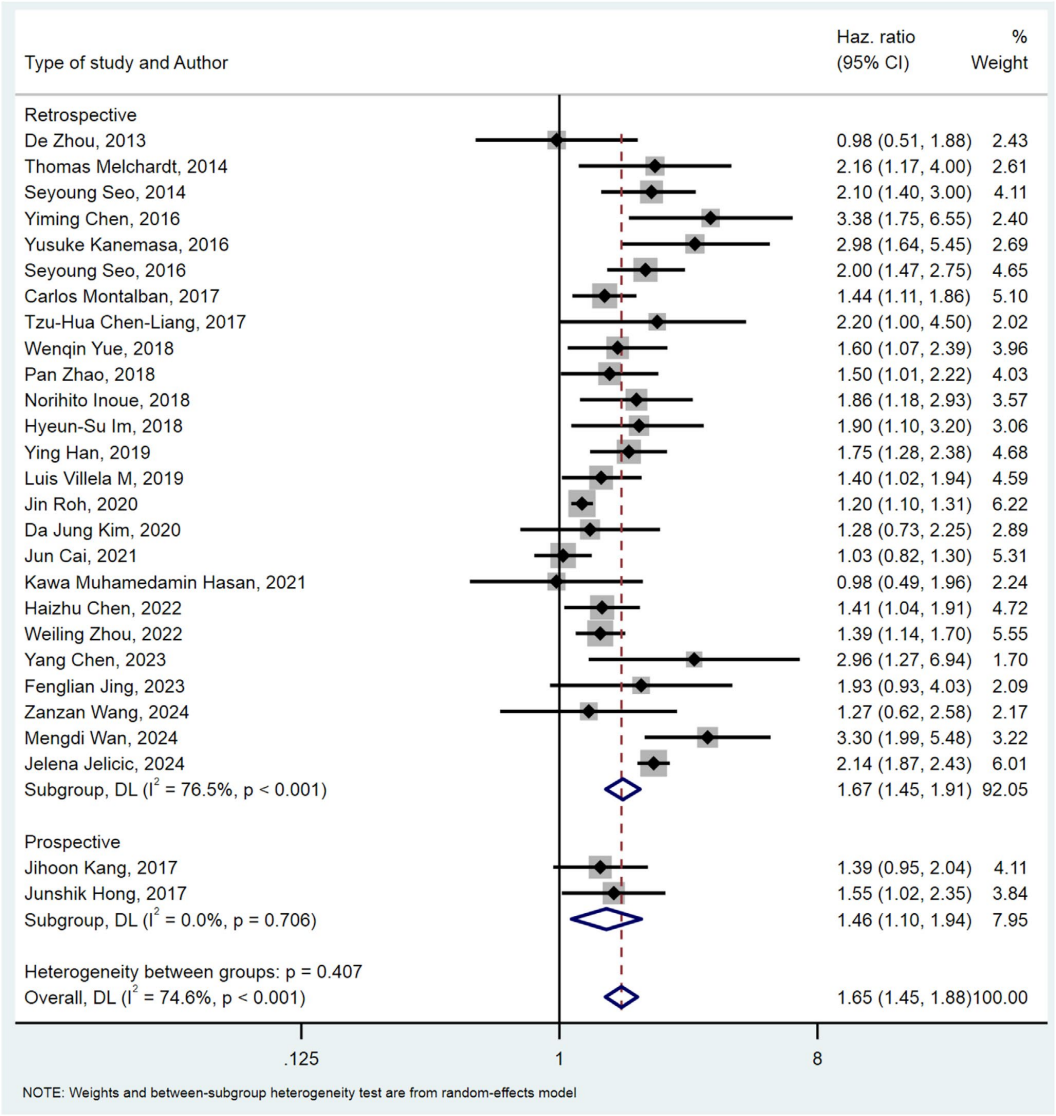
Forest plot of pooled HR for OS in relation to β2M.

In total, 26 studies [19, 20, 21, 22, 26, 27, 28, 29, 30, 34, 35, 36, 37, 38, 39, 40, 41, 42, 43, 46, 47, 48, 49, 50, 51, 52, 53] with a combined sample size of 17 407 patients analyzed the relationship between OS and β2M in DLBCL patients. The meta‐analysis demonstrated a significant association between OS and β2M levels in DLBCL patients. Heterogeneity analysis revealed significant heterogeneity among the studies (*I* ^2^ = 74.6%, *p*‐value < 0.01). Therefore, a random‐effects model was used for analysis and pooled HR calculation. Pooled analysis results showed that higher β2M levels in DLBCL patients were associated with worse prognosis and lower OS (HR _Pooled_: 1.65, 95% CI: 1.45–1.88, *p*‐value < 0.01) (Figure 2).

### 3.2 Relationship Between PFS and β2M

**FIGURE 3:**
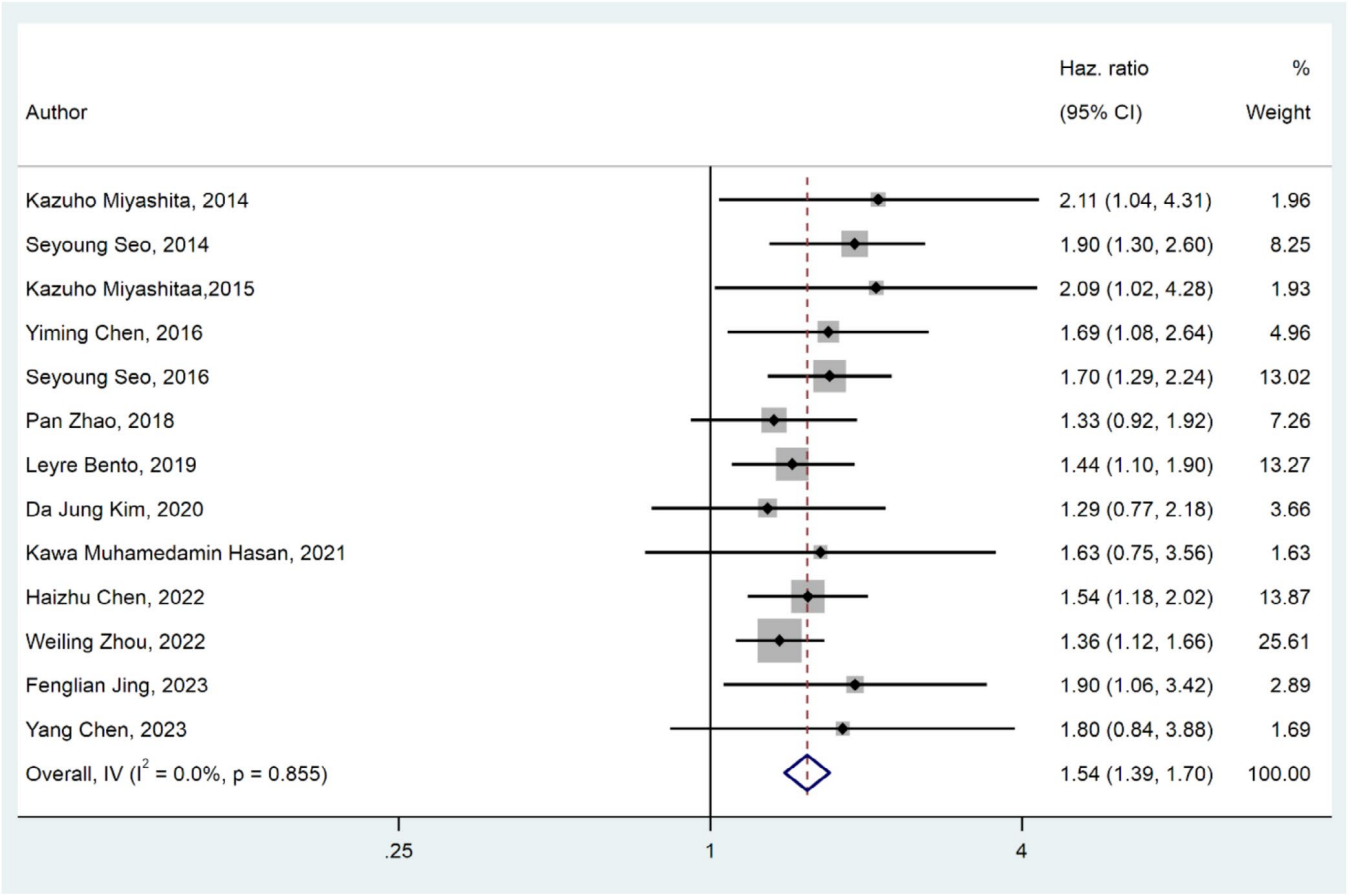
Forest plot of pooled HR for PFS in relation to β2M.

In total, 13 studies [16, 19, 21, 28, 29, 35, 41, 44, 45, 47, 48, 51, 53] with a combined sample size of 7721 patients studied the relationship between PFS and β2M in DLBCL patients, revealing a significant correlation between PFS and β2M in DLBCL patients. Heterogeneity analysis showed no significant heterogeneity among the studies (*I* ^2^ = 0%, *p*‐value = 0.855). Therefore, a fixed‐effects model was used. Pooled analysis results indicated that higher β2M levels in DLBCL patients were associated with shorter PFS (HR _Pooled_: 1.54, 95% CI: 1.39–1.70, *p*‐value < 0.01) (Figure 3).

### 3.3 Sensitivity Analysis

To assess the impact of each included study on the overall effect size in this meta‐analysis, leave‐one‐out sensitivity analysis was performed. The results indicated no significant variation among the included studies regarding the relationship between OS and PFS with β2M (Supporting Information S2).

### 3.4 Meta‐Regression Analysis

**FIGURE 4:**
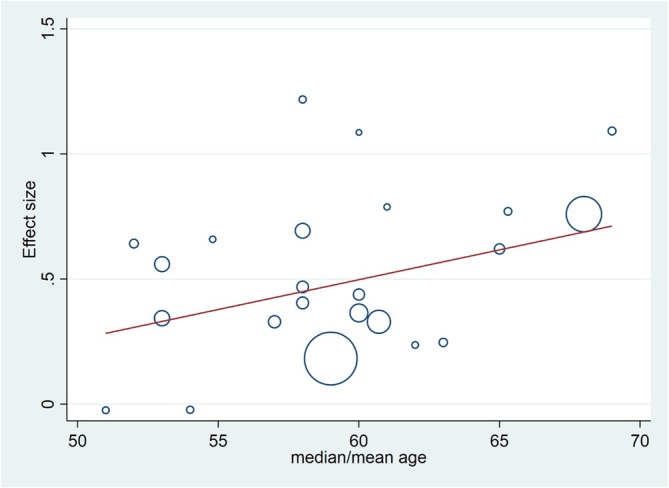
Meta‐regression analysis of effect size and age in OS analysis.

The analysis of the relationship between other variables and overall effect size showed no significant association between mean/median age, follow‐up duration, or gender ratio with effect size in OS analysis (*p*‐value > 0.05) (Figure 4). Similarly, no significant association was found in PFS analysis (*p*‐value > 0.05) (Supporting Information S1: Table 2).

### 3.5 Publication Bias

**FIGURE 5:**
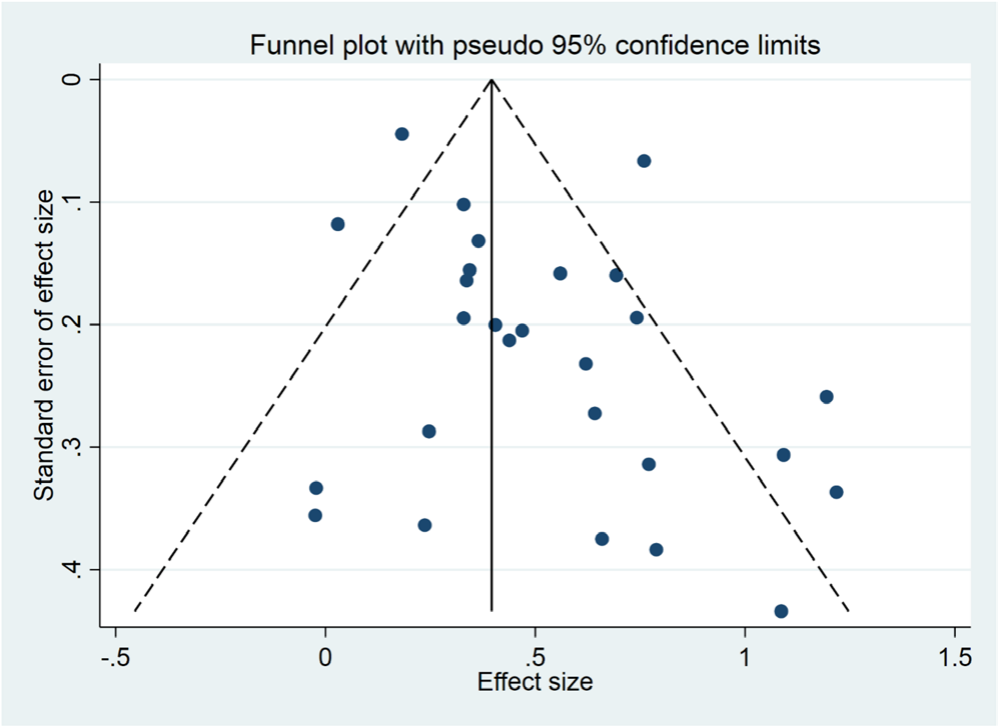
Funnel plot of pooled HR for OS.

**FIGURE 6:**
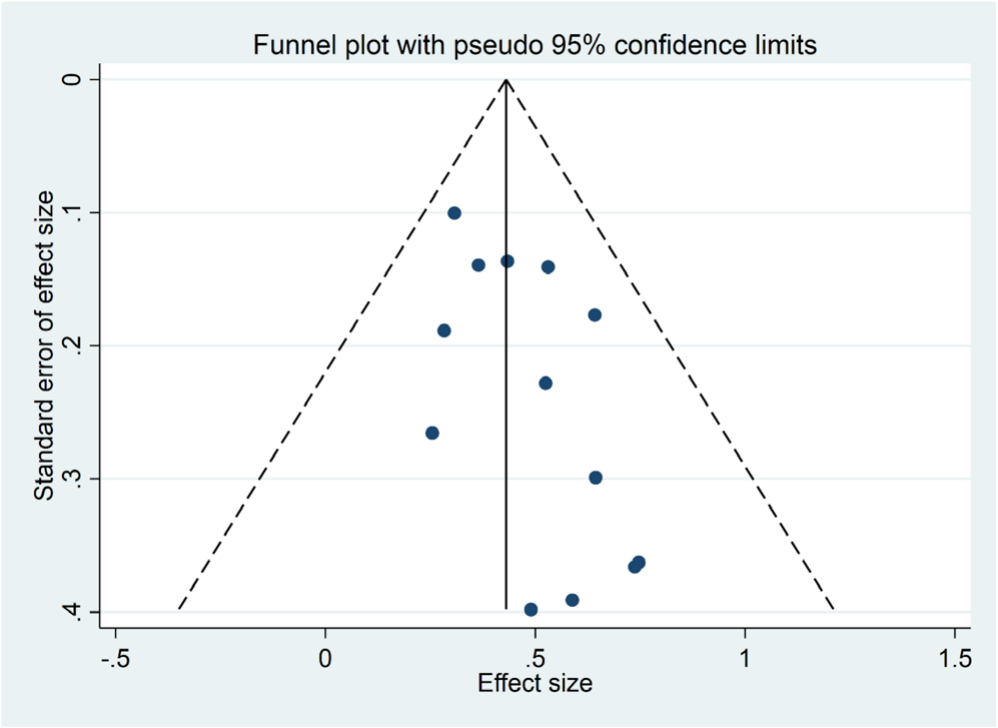
Funnel plot of pooled HR for PFS.

Publication bias regarding the relationship between β2M and OS and PFS was assessed using funnel plot symmetry analysis, indicating relative symmetry (Figures 5 and 6). Additionally, Egger's test and Begg's test confirmed no significant publication bias in the meta‐analysis (*p*‐value > 0.05) (Supporting Information S1: Table 3).

## 4 Discussion

In this meta‐analysis, by aggregating existing studies, the prognostic significance of β2M for OS and PFS was examined for patients with DLBCL. The pooled analysis results clearly demonstrated that β2M serves as an independent prognostic factor associated with OS and PFS in DLBCL patients.

Recent evidence strongly suggests that β2M can act as an independent prognostic factor for various types of Hodgkin and non‐Hodgkin lymphomas, including aggressive and indolent lymphomas [54, 55, 56], mantle cell lymphoma [57], extranodal natural killer/T‐cell lymphoma [55, 58], and follicular lymphoma [59, 60]. However, the high heterogeneity and poor prognosis of DLBCL patients, which greatly impact the disease's clinical course, have made precise risk stratification increasingly essential. Recent studies have focused on the prognostic role of β2M in DLBCL patients to improve risk classification models. Several large‐scale and retrospective studies have highlighted the prognostic significance of β2M in DLBCL patients [19, 21, 22, 26, 27]. However, some studies have not found evidence supporting the prognostic role of β2M in DLBCL patients [28, 29, 30]. The findings of this meta‐analysis also indicated that elevated β2M levels are associated with poorer prognosis, including reduced OS and PFS in DLBCL patients.

β2M is a small, lightweight polypeptide chain that is a component of the HLA class I complex, widely distributed in nucleated cells, particularly in immune cells such as lymphocytes and monocytes [61]. The kidneys play a major role in the metabolism and excretion of β2M [44]. Although the precise mechanism of β2M as a prognostic factor in DLBCL remains unclear, its serum levels reflect its release from the membranes of white blood cells (its primary source) and the cytoplasm of cells, correlating with cell proliferation, thereby serving as a tumor burden marker [62]. Additionally, studies have linked elevated β2M levels with the development of more aggressive lymphoid malignancies, increased tumor burden, and higher cellular turnover rates [16]. Since β2M is eliminated through the kidneys, renal damage or failure can lead to increased β2M levels, similar to conditions involving systemic inflammation, cardiovascular diseases, or the aging process [61]. Josson et al. [63] demonstrated the role of β2M in cancer metastasis and lethality, showing that β2M is involved in key biological processes such as growth and apoptosis signaling pathways, making it a potential therapeutic target.

Our study further confirms that elevated β2M levels are linked to poorer OS and PFS in DLBCL patients. Tumor burden and cellular turnover are additional factors that may explain the prognostic role of β2M in cancer patients. Shi et al. [62] reported that β2M‐specific antibodies exhibit significant tumoricidal activity against both hematologic and non‐hematologic malignancies, selectively targeting cancer cells while sparing normal cells, highlighting its potential clinical significance.

In a study conducted by Melchardt et al. [43] on 499 DLBCL patients, the validity of the NCCN‐IPI was confirmed in a European population, demonstrating an association between β2M and patient prognosis. Overall, β2M provides valuable prognostic information to NCCN‐IPI, enhancing its clinical significance. Similarly, Kanemasa et al. [22] observed the prognostic role of β2M in Japanese DLBCL patients, suggesting that β2M, along with age, performance status, and Ann Arbor stage, offers superior risk stratification compared to NCCN‐IPI.

Although β2M is a strong prognostic factor across different histological subtypes of lymphoma, it has not yet been incorporated into prognostic models for lymphoma, except for follicular lymphoma [60]. Current evidence suggests that β2M could enhance the accuracy of prognostic models for DLBCL. Some studies have proposed new risk models based on the role of β2M in patient survival and prognosis, potentially improving or even outperforming NCCN‐IPI, particularly in patients treated with rituximab and other therapeutic regimens [16, 19, 21, 27, 42].

In this meta‐analysis, the lack of significant differences in treatment regimens among DLBCL patients limited the ability to thoroughly assess the role of β2M in relation to specific chemotherapy protocols. Nonetheless, the pooled analysis confirmed the prognostic significance of β2M for OS and PFS in DLBCL patients. Given that β2M can be easily and cost‐effectively measured from routine patient tests, its incorporation into risk classification models may offer valuable clinical insights. However, this study has several limitations. Most included studies were retrospective, which limited analyses by study type and introduced potential biases, including information bias, that should be carefully considered when interpreting and applying the findings. Additionally, baseline cut‐off values used to categorize patients based on β2M levels varied considerably across studies, precluding subgroup analysis. While meta‐regression was conducted to assess the impact of other key variables, the lack of comprehensive and consistent data restricted more detailed subgroup analyses. A notable limitation of this meta‐analysis is the variability in cut‐off values for β2M across included studies. Diverse laboratory normal values and thresholds used to define elevated β2M levels may contribute to heterogeneity and influence pooled prognostic estimates. Furthermore, variations in laboratory reference ranges resulting from differing assay methods or population characteristics may introduce bias and limit direct comparability of results. Future research should focus on establishing standardized cut‐off values for β2M to improve consistency and clinical applicability in prognostic assessments.

## 5 Conclusion

This meta‐analysis evaluated the prognostic significance of β2M in patients with DLBCL and demonstrated that elevated serum β2M levels are significantly associated with poorer PFS and OS. Due to its accessibility, cost‐effectiveness, and feasibility of measurement through routine CBC tests, β2M represents a practical biomarker for clinical use. Therefore, incorporating β2M into established prognostic models, such as the NCCN‐IPI, is recommended to improve risk stratification accuracy and enhance the predictive validity of outcomes in DLBCL patients. Integrating β2M assessment into routine clinical practice could facilitate more personalized management by identifying high‐risk individuals who may benefit from more aggressive or alternative therapeutic strategies, ultimately improving patient prognosis and care.

## Author Contributions

M.K., K.R., and H.F.D. contributed to the design and implementation of the study. R.S. contributed to the analysis and interpretation of the data. H.F.D. and M.R. contributed to the assessment of the quality of the studies. M.M., R.S., M.F., and K.R. contributed to the interpretation of the data and participated in drafting the manuscript. K.R., V.S.S., and R.S. contributed to data extraction and management. All authors read and approved the final manuscript.

## Funding

The authors have nothing to report.

## Conflicts of Interest

The authors declare no conflicts of interest.

## Supporting information


**Table S1:** Search strategy for prognostic value of Beta‐2 Microglobulin in DLBCL patients.
**Table S2:** Meta regression analysis for OS and PFS.
**Table S3:** Publication bias in included studies.

**Figure S1:** Sensitivity analysis in relevance of β2M and OS.
**Figure S2:** Sensitivity analysis in relevance of β2M and PFS.

## Data Availability

The data that support the findings of this study are available on request from the corresponding author. The data are not publicly available due to privacy or ethical restrictions.
