# Supplementary material for: Prognostic Role of Beta‐2 Microglobulin in Diffuse Large B‐Cell Lymphoma: Systematic Review and Meta‐Analysis of Observational Studies
Source: Cancer Rep (Hoboken). 2025 Nov 29;8(12):e70416. doi: 10.1002/cnr2.70416 (PMC12664714; doi:10.1002/cnr2.70416)
Supplement: Supplementary file 1 — Table S1: Search strategy for prognostic value of Beta‐2 Microglobulin in DLBCL patients. Table S2: Meta regression analysis for OS and PFS. Table S3: Publication bias in included studies. [file CNR2-8-e70416-s001.docx]

**S1 Table:** Search strategy for prognostic value of Beta-2 Microglobulin in DLBCL patients.

| **Databases** | **Search terms** |
| --- | --- |
| PubMed/MEDLINE, Web of Science, Scopus, | (**Beta 2-****Microglobulin**) OR (**β 2-Microglobulin**) OR (**β2M**) OR (**Beta 2 M**) and “**DLBCL**” or **Diffuse large B-cell lymphoma** or **Diffuse large B cell** or **B cell lymphoma or cancer or lymphoma or leukaemia”**. |
| **Number of papers identified** | **Results in each database** |
| 852 journal papers | PubMed/Medline=252 papers  Scopus=427 papers  Web of Science=173 papers |

**S2 Table - Meta regression analysis for OS and PFS**

| **P value** | **SE** | **Coefficient** | **Variables** | **Type of analysis** |
| --- | --- | --- | --- | --- |
| 0.053 | 0.011 | 0.023 | **Median/mean age** | **OS analysis** |
| 0.243 | 0.334 | -0.401 | **Gender ratio (male/female)** |  |
| 0.460 | 0.0036 | -0.0027 | **Median follow-up time** |  |
| 0.533 | 0.014 | -0.0094 | **Median/mean age** | **PFS analysis** |
| 0.398 | 0.259 | 0.228 | **Gender ratio (male/female)** |  |
| 0.581 | 0.0032 | 0.0019 | **Median follow-up time** |  |

**S3 Table - Publication bias in included studies**

| **Publication Bias** | | **β/ Kendall's score** | **SE beta/ score** | **Z** | **P value** |
| --- | --- | --- | --- | --- | --- |
| **OS analysis** | **Egger test** | 1.06 | 0.668 | 1.59 | 0.112 |
|  | **Begg test** | 59 | 47.969 | 1.21 | 0.226 |
| **PFS analysis** | **Egger test** | 0.97 | 0.669 | 1.45 | 0.146 |
|  | **Begg test** | 18 | 16.39 | 1.04 | 0.299 |
