# Supplementary material for: Prognostic Role of Beta‐2 Microglobulin in Diffuse Large B‐Cell Lymphoma: Systematic Review and Meta‐Analysis of Observational Studies
Source: Cancer Rep (Hoboken). 2025 Nov 29;8(12):e70416. doi: 10.1002/cnr2.70416 (PMC12664714; doi:10.1002/cnr2.70416)
Supplement: Supplementary file 2 — Figure S1: Sensitivity analysis in relevance of β2M and OS. Figure S2: Sensitivity analysis in relevance of β2M and PFS. [file CNR2-8-e70416-s002.docx]

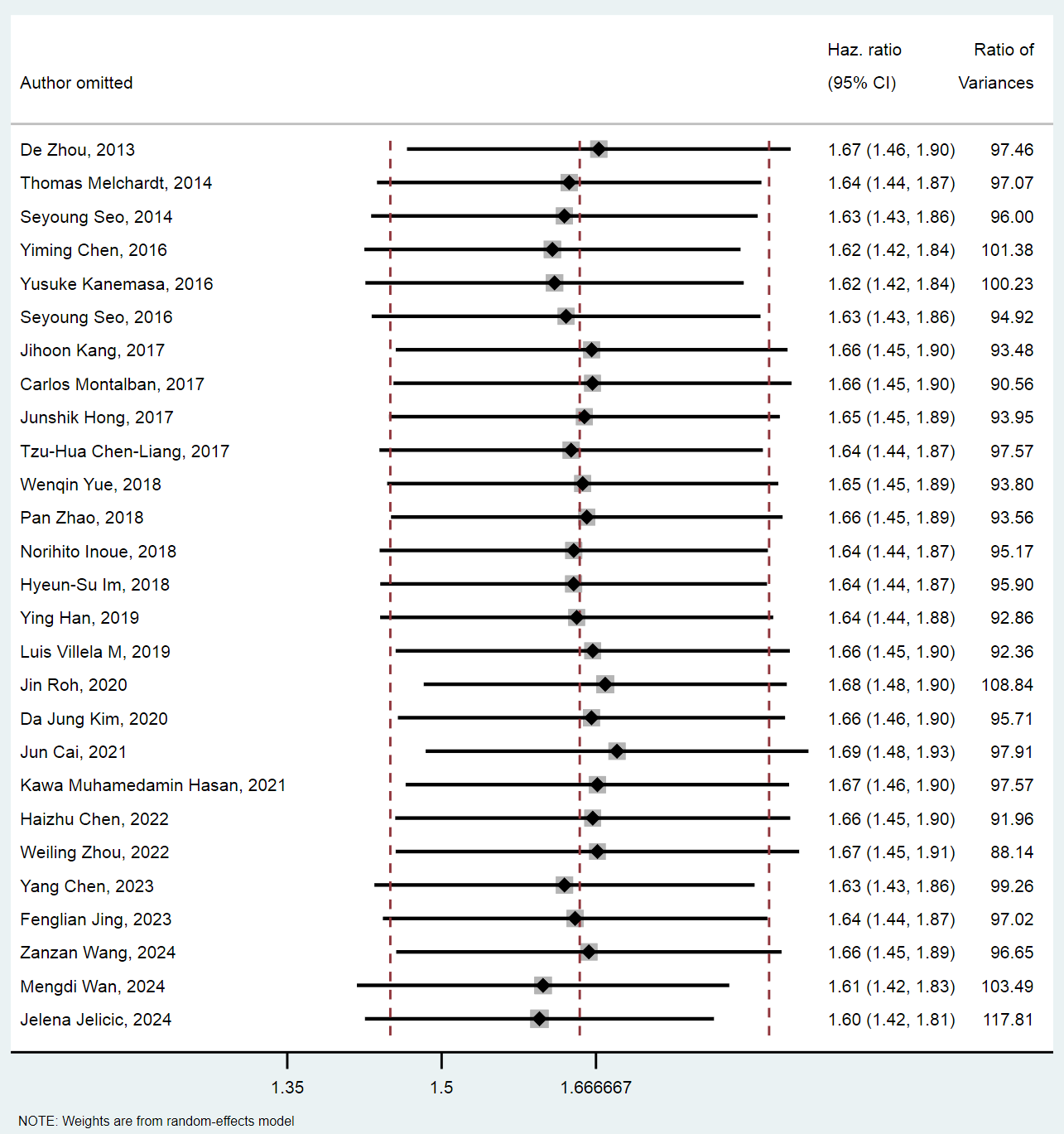


Fig 1- Sensetivity analysis in relevanve of β2M and OS


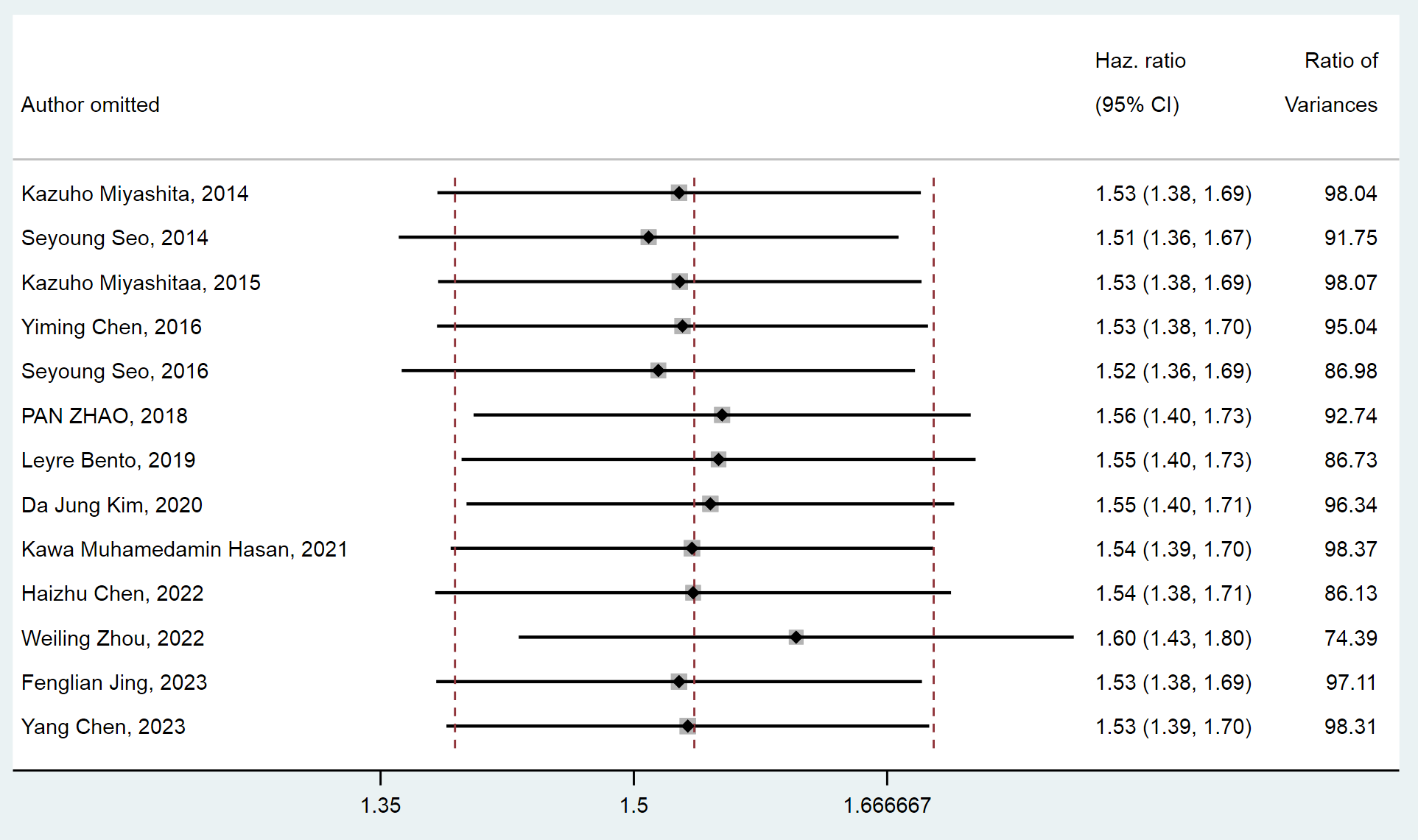


Fig 2- Sensetivity analysis in relevanve of β2M and PFS
